# Oral Bacterial Microbiota in Digestive Cancer Patients: A Systematic Review

**DOI:** 10.3390/microorganisms9122585

**Published:** 2021-12-14

**Authors:** Elisa Reitano, Nicola de’Angelis, Paschalis Gavriilidis, Federica Gaiani, Riccardo Memeo, Riccardo Inchingolo, Giorgio Bianchi, Gian Luigi de’Angelis, Maria Clotilde Carra

**Affiliations:** 1 Division of General Surgery, Department of Translational Medicine, Maggiore della Carità Hospital, University of Eastern Piedmont, 28100 Novara, Italy; elisa.reitano@live.it; 2 Unit of Digestive and HPB Surgery, CARE Department, Henri Mondor Hospital, AP-HP, 94010 Créteil, France; nic.deangelis@yahoo.it (N.d.); giorgio.bianchi@gmail.com (G.B.); 3 Faculté de Santé, Université Paris Est, UPEC, 94010 Créteil, France; 4 Department of HBP Surgery, University Hospitals Coventry and Warwickshire NHS Trust, Clifford Bridge Road, Coventry CV2 2DX, UK; pgavrielidis@yahoo.com; 5 Gastroenterology and Endoscopy Unit, Department of Medicine and Surgery, University Hospital of Parma, 43126 Parma, Italy; federica.gaiani@unipr.it; 6 Microbiome Research Hub, University of Parma, 43126 Parma, Italy; 7 Unit of HPB Surgery, General Regional University Hospital F. Miulli, Acquaviva delle Fonti, 72021 Bari, Italy; drmemeo@yahoo.it; 8 Unit of Interventional Radiology, General Regional Hospital F. Miulli, Acquaviva delle Fonti, 72021 Bari, Italy; riccardoin@hotmail.it; 9 Service of Odontology, Department of Periodontology, Rothschild Hospital, AP-HP, Université de Paris, U.F.R. of Odontology-Garanciere, 75006 Paris, France; mclotildecarra@gmail.com

**Keywords:** oral microbiota, carcinogenesis, digestive cancer, systematic review

## Abstract

The relation between the gut microbiota and human health is increasingly recognized. Recently, some evidence suggested that dysbiosis of the oral microbiota may be involved in the development of digestive cancers. A systematic review was conducted according to the PRISMA guidelines to investigate the association between the oral microbiota and digestive cancers. Several databases including Medline, Scopus, and Embase were searched by three independent reviewers, without date restriction. Over a total of 1654 records initially identified, 28 studies (2 prospective cohort studies and 26 case-controls) were selected. They investigated oral microbiota composition in patients with esophageal squamous cell carcinoma (*n* = 5), gastric cancer (*n* = 5), colorectal cancer (*n* = 9), liver carcinoma (*n* = 2), and pancreatic cancer (*n* = 7). In most of the studies, oral microbiota composition was found to be different between digestive cancer patients and controls. Particularly, oral microbiota dysbiosis and specific bacteria, such as *Fusobacterium nucleatum* and *Porphyromonas gingivalis*, appeared to be associated with colorectal cancers. Current evidence suggests that differences exist in oral microbiota composition between patients with and without digestive cancers. Further studies are required to investigate and validate oral–gut microbial transmission patterns and their role in digestive cancer carcinogenesis.

## 1. Introduction

Digestive cancers include cancers located in the esophagus, stomach, liver, pancreas, colon, and rectum. Their incidence and related mortality are increasing worldwide, but with some characteristic geographical differences [1]. According to the GLOBOCAN, i.e., Cancer Incidence in Five Continents, and the World Health Organization (WHO) mortality databases in 2018, the majority of new cases of digestive cancers (63%) and related deaths (65%) occurred in Asia, followed by Europe and North America. Moreover, esophageal, gastric, and liver cancers appear to be more prevalent in Asia, whereas colorectal and pancreatic cancers are more common in Europe and North America [2]. Most of these cancers are considered sporadic and are influenced by several potentially modifiable environmental factors, such as tobacco smoking, diet, alcohol consumption, physical inactivity, obesity, and immunosuppressive drugs [1]. Some recent evidence suggested a role of the human microbiota in the development of digestive cancers, not only related to the composition and changes in relative abundance of microbes of the gut microbiota [3,4,5] but also linked to a state of dysbiosis of the oral microbiota [6,7]. In this review, we define “dysbiosis” as any change to the composition of resident bacterial communities relative to the community found in healthy individuals [8].

The oral cavity is a reservoir of more 700 species or phylotypes of bacteria, of which approximately 35% have not been cultured yet [9]. The equilibrium of this complex ecosystem is essential for oral health and influences host responses to disease [10]. The disruption in the homeostasis, i.e., dysbiosis, can result in significant metabolic and immunologic effects on the host, ultimately leading to local and systemic diseases [11,12]. These mechanisms are well documented in the pathogenesis of periodontitis, a chronic multifactorial inflammatory disease of the tooth-supporting tissues including the gums, the periodontal ligament, and the alveolar bone [13], but it has also been observed for cardiovascular, neurological, and metabolic disorders as well as digestive cancers and inflammatory bowel diseases [7,13,14,15].

In particular, specific oral bacteria that are typically found in the oral cavity of individuals suffering from periodontal diseases, such as *Fusobacterium nucleatum* [16] and *Porphyromonas gingivalis* [17], have been found in significantly high abundance in tumoral tissues and fecal samples of patients affected by colorectal cancer (CRC) [18,19], supporting the ability of these bacteria to migrate through the gastrointestinal tract where they can induce inflammation, alter the host immune response, and create an environment that may eventually favor tumor growth [20,21,22,23]. Specifically, data from a mouse model of intestinal tumorigenesis suggest that fusobacteria generate a proinflammatory microenvironment that is conducive to CRC progression through recruitment of tumor-infiltrating immune cells [24,25,26]. This was confirmed in clinical studies analyzing *Fusobacterium nucleatum* abundance by quantitative real-time polymerase chain reaction of DNA extracted from colorectal tissue biopsies and surgical resection specimens and observing that *Fusobacterium nucleatum* was more abundant in stool samples from CRC patients compared with adenomas or controls [27,28,29]. Some authors concluded that *Fusobacterium nucleatum* could be a novel risk factor for disease progression from adenoma to cancer, possibly affecting patient survival outcomes [27].

In this perspective, recent evidence suggested that certain oral bacteria and in general the characterization of the oral microbiota composition may be used as biomarkers for the detection of certain digestive cancers, with a potential role as a non-invasive screening tool [18,30,31].

The present systemic review aims to provide an updated appraisal of the current literature investigating the association between the oral microbiota and different types of digestive cancers, by (i) describing the observed differences in oral bacterial composition and abundance in cancer patients vs. controls and (ii) suggesting the possible impact of oral microbiota on digestive cancer risk.

## 2. Materials and Methods

### 2.1. Protocol Development and Literature Search

The present systematic review was performed according to the Cochrane collaboration-specific protocol [32] and was reported following the Preferred Reporting Items for Systematic Reviews and Meta-analyses (PRISMA) checklist (Table S1) [33].

Studies that investigated the association between oral microbiota and digestive cancers were searched in the following databases without date restrictions: Medline (through PubMed), Scopus, Embase, Cochrane Library and Google Scholar.

A specific research equation was used for each database, using the following keywords and MeSH terms: oral microbiome, oral microbiota, mouth microbiome, gastrointestinal neoplasms, gastrointestinal cancers, gastrointestinal carcinoma, digestive cancer, digestive neoplasms.

According to the PICOS schema, the following criteria were used to construct the literature search and to select the pertinent articles:

Population: adult patients with a diagnosis of digestive cancer (all types of cancers located in the digestive apparatus).
Intervention: analysis of oral microbiota composition and/or oral microbiome with or without a concomitant oral/periodontal examination. No restriction was applied for the type of microbiological technique used to sample and analyze the oral microbiota/microbiome, which include culture-dependent and genome sequencing methods.
Comparison(s): adult patients without cancer.
Outcome(s): oral microbiota composition (quality and quantity of bacterial species and pathogens related to oral diseases, particularly periodontitis).
Study designs: All types of descriptive (case series, cross-sectional) and analytic studies (cohort, case-control, clinical trial) estimating the magnitude of the association between oral microbiota dysbiosis and digestive cancers.

Results were limited to human clinical studies with review articles, with case reports being excluded. The literature review was completed by an extensive search using the “related articles” function in PubMed. The reference lists of the eligible records and of review articles were double-checked to identify potential additional pertinent articles. Articles were selected and reviewed if written in English only.

The literature search and selection were performed independently and blindly by three reviewers (E.R., G.B. and M.C.C.). Records were removed from the selection if all reviewers excluded the articles at the title/abstract screening levels. Disagreements were resolved with discussion with a third reviewer (N.d.A.).

### 2.2. Data Extraction

The reviewers performed an independent full-text analysis and data extraction by filling an electronic database. Extracted data included first author name, country, journal, year of publication, number of patients, age of the patients, type of cancer, oral diagnosis, type of microbiota analysis, sample extraction, detection method, and main results.

### 2.3. Study Quality Assessment and Risk of Bias

The reviewers also carried out a study quality assessment and risk of bias evaluation of the selected articles. According to the type of study design, the Newcastle–Ottawa scale [34] (NOS) was used.

## 3. Results

### 3.1. Literature Search and Selection

**Figure 1:**
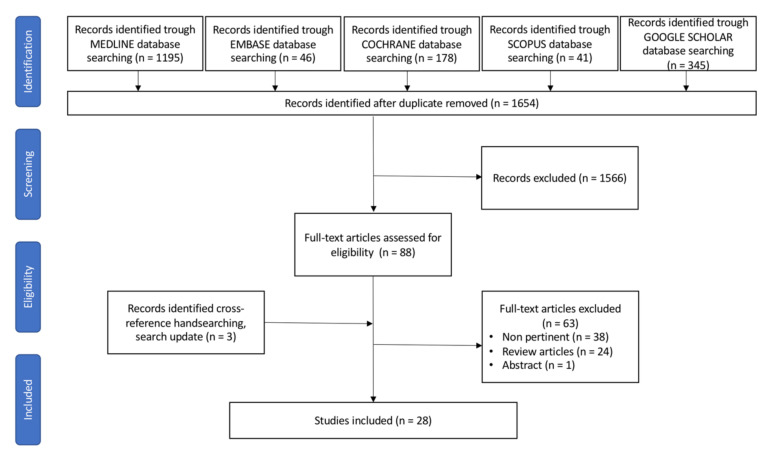
PRISMA flow diagram for study search, selection, inclusion, and exclusion. Example of the research strategy: (oral microbiota[Title/Abstract]) OR (oral microbiome[Title/Abstract]) OR (mouth microbiota[Title/Abstract]) OR (mouth microbiome[Title/Abstract]) OR (oral cavity[Title/Abstract]) OR (oral bacteria[Title/Abstract]) AND (digestive cancer[Title/Abstract]) OR (digestive[Title/Abstract]) OR (digestive neoplasm[Title/Abstract]) OR (cancer of digestive system[MeSH Terms]) OR (cancer of the digestive system[MeSH Terms]) OR (cancer, digestive system[MeSH Terms]) OR (cancer of gastrointestinal tract[MeSH Terms]) OR (cancer of the gastrointestinal tract[MeSH Terms]) OR (gastrointestinal neoplasm[Title/Abstract]) OR (gastrointestinal cancer[Title/Abstract]).

The merged search yielded 1805 results. After removing duplicates, 1654 articles were screened for eligibility based on title and abstract. Twenty-eight articles fulfilled the inclusion criteria and were selected for the present systematic review, as shown in Figure 1.

### 3.2. Studies Characteristics

The selected studies were published between 2012 and 2021. There were 2 prospective cohort studies and 26 case-control studies (of which 6 were based on matched case-control groups). The studies were carried out in Europe (*n* = 3), USA (*n* = 8), China (*n* = 14), Japan (*n* = 1), Turkey (*n* = 1), and Iran (*n* = 1). The general characteristics of the studies examined are summarized in Table 1. Concerning the type of cancers, nine studies (31%) investigated oral microbiota composition in patients with colorectal cancer (CRC) [3,16,35,36,37,38,39,40,41], seven on pancreatic cancer (PC) [30,42,43,44,45,46,47], six on gastric cancer (GC) [39,48,49,50,51,52], five on esophageal cancer (EC) [6,53,54,55,56], and two on liver cancer (LC) [57,58].

All studies described microbiota composition differences between cases (cancer patients) and controls, and several studies [6,39,40,41,47,49,50,56,57] also analyzed the oral microbiota as being possible biomarkers for cancer screening and early diagnosis. The overall total number of digestive cancer patients considered was 2708, who were compared with 2593 controls. Twenty-six studies used DNA analysis, with 16S rRNA V4 sequencing and PCR as the principal method for microbiota investigation, whereas indirect immunofluorescence was used in one study [36] and shotgun sequence in another one [38]. Study outcomes are displayed in Table 2.

### 3.3. Oral Microbiota and Esophageal Cancer (EC)

Five articles investigated the association between oral microbiota and EC [6,53,54,55,56].

Chen et al. [53] demonstrated that patients with esophageal squamous cell carcinoma (ESCC) have a decreased oral microbiota diversity when compared with non-ESCC controls. The authors described a decreased salivary carriage of genera *Lautropia*, *Bulleidia*, *Catonella*, *Corynebacterium*, *Moryella*, *Peptococcus*, and *Cardiobacterium* and higher relative abundance of *Prevotella*, *Streptococcus*, and *Porphyromonas* in the ESCC group [53]. Wang et al. [54] showed that *Actinomyces* and *Antopobium* were related to a higher risk of ESCC, whereas the healthy group of the study was closely related to *Fusobacterium* and *Porphyromonas*. Zhao et al. [55] showed that ESCC is associated with an increased salivary carriage of *Firmicutes*, *Negativicutes*, *Selenomonadales*, *Prevotellaceae*, *Prevotella*, and *Veillonellaceae* and with a decreased taxa of *Proteobacteria*, *Betaproteobacteria*, *Neisseriales*, *Neisseriaceae*, and *Neisseria*.

Kawasaky et al. and Peters et al. concluded that differences in microbiota compositions between cases and controls could be used as a possible biomarker for cancer screening [6,56]. Only one study investigated the microbiota in relation to an esophageal adenocarcinoma, showing an association with an increased oral rinse carriage of *Tannarella forsythia* and a depletion of the commensal genus *Neisseria* and *Streptococcus Pneumoniae* [6].

### 3.4. Oral Microbiota and Liver Cancer (LC)

Only two articles investigated the association between oral microbiota dysbiosis, and LC. Lu et al. [57] identified *Oribacterium* and *Fusobacterium* as possible biomarkers to identify LC patients, showing a significant difference in their relative abundance between healthy controls and LC patients [57]. Li et al. compared the oral microbiota of patients with LC with healthy controls and patients with cirrhosis in different stages, finding an association between cancer and *Haemophilus*, *Porphyromonas*, and *Filifactor*.

### 3.5. Oral Microbiota and Pancreatic Cancer (PC)

In the seven studies dealing with PC, the microbiota analysis was conducted on salivary samples in five studies [30,42,43,44,47], on tongue coating in one study [46], and on oral rinse [45] in the remaining study. Results were heterogeneous. Lu et al. as well as Fan et al. observed an increased risk of cancer linked to *Porphyromonas gingivalis*, but contrasting findings were reported in relation to *Fusobacterium*, which was respectively linked to an increased and decreased risk of cancer [45,46]. The remaining studies found different bacteria linked to PC risk, without highlighting the predominance of a specific microbiota.

### 3.6. Oral Microbiota and Gastric Cancer (GC)

The microbiota analysis was conducted on tongue coating in four studies [39,48,51,52] and on salivary sample in two studies [49,50]. Hu et al. [48], Xu et al. [52], and Han et al. [39] reported that tongue coating characteristics significantly differ between cases and controls, with thicker tongue coating associated with an increased risk of GC. Xu et al. [52] divided the sample of GC patients into five subgroups based on the tongue coating characteristics, reporting different microbial associations in the different groups. However, also for this type of cancer there was no evidence of a specific microbiota being related to the development of GC.

### 3.7. Oral Microbiota and Colorectal Cancer (CRC)

Microbial samples were collected by oral swab in three studies [3,16,40], oral rinse in one study [45], saliva in three studies [36,38,41], subgingival plaque in one study [35], and tongue coating in one study [39]. Overall, these studies showed the most consistent results. *Fusobacterium nucleatum* was found to be linked to an increased risk of CRC in five studies [35,36,39,40,41]. Four studies agreed on the association of different species of *Prevotella* and CRC risk [3,35,37,40], while a borderline association was found in one study [39].

### 3.8. Study Quality Assessment

Based on the NOS assessment [34], 4 studies had a score of 5/9 [16,38,47,54], 16 studies of 6/9 [3,6,30,36,37,41,42,46,48,49,51,52,53,55,57,58], and 8 studies of 7/9 [35,39,40,43,44,45,50,56] (Table 3). Globally, a high heterogeneity was observed in study designs, study populations, and oral microbiota evaluation methods.

## 4. Discussion

The present systematic review provides a synthesis of studies investigating the association between oral microbiota composition and digestive cancers with a systematic approach. The available evidence suggests that digestive cancer patients present an oral microbiota composition that differs from non-cancer controls and specific oral bacteria may be linked to increased odds for digestive cancers. The predominance and/or relative abundance of these bacteria could have a synergistic effect on digestive cancers’ etiology [3,6,36,37,40,44,45,49,51,55], suggesting a possible role of oral microbiota characterization for screening and risk assessment of some types of cancer [40,47,49,56,59]. However, it is important to highlight that the current body of evidence is still limited, and some digestive cancers are under-investigated. For instance, only two studies were found concerning LC [57,58]. More studies are available concerning the role of oral microbiota in PC, GC, EC, and CRC patients, but further research is needed before advancing any solid conclusion. Notwithstanding this, the overall body of evidence consistently suggests that the oral microbiota is linked to the risk of digestive cancers.

In the digestive tract, the microbiota synthetizes essential amino acids and vitamins taking part in the digestion process. A symbiotic microbiota is normally characterized by a high diversity, and it is able to resist changes that occur during physiological stress [60], and to stimulate an immune response [20]. In recent years, different studies focused on the possible role of intestinal and oral microbiota dysbiosis in disease development, especially in carcinogenesis [7,20,23,53]. The digestive tract microbiota is involved in inflammation, metabolism, and genotoxicity mechanisms taking part in the oncogenic process [60]. Meanwhile, a moderate inflammatory reaction is protective against cancer development, and excessive inflammatory response is correlated with a higher risk of carcinogenesis [59]. Different oral bacteria associated with carcinogenesis, such as *Porphyromonas*, *Prevotella*, and *Fusobacterium*, can contribute to a chronic inflammatory reaction by stimulating the production of inflammatory mediators such as interleukin-1β, interleukin-6, and matrix metalloproteinases [61,62]. *Prevotella* and *Fusobacterium* have also been linked to carcinogenesis by the mechanisms of suppressing host immunological functions and promoting the malignant transformation of epithelial cells [28,29,59].

The potential translocation ability of the oral microbiota through the circulation system (hematogenous route) or following the flow of food and fluids into the digestive system (enteral route) could explain its presence in the gut and its role in carcinogenesis [59]. Three possible mechanisms of bacterial translocation in animal models were suggested: (1) disruption of the digestive equilibrium with intestinal bacterial overgrowth, (2) increased permeability of the intestinal mucosal barrier, and (3) deficiencies in host immune defenses. However, the transmission pathway remains questionable, and no definitive reports are currently available on the topic [63]. Zhang et al. [40] conducted an additional functional analysis using PICRUSt (Phylogenetic Investigation of Communities by Reconstruction of Unobserved States) and showed that the membrane transport pathway was decreased in CRA and CRC patients compared with healthy controls, with a potential impact on the anti-tumor immune response (such as the response mediated by bacterial outer membrane vesicles). Moreover, the cell motility pathway was found to be overrepresented in cancer patients, promoting tumor invasion and migration [40].

**Figure 2:**
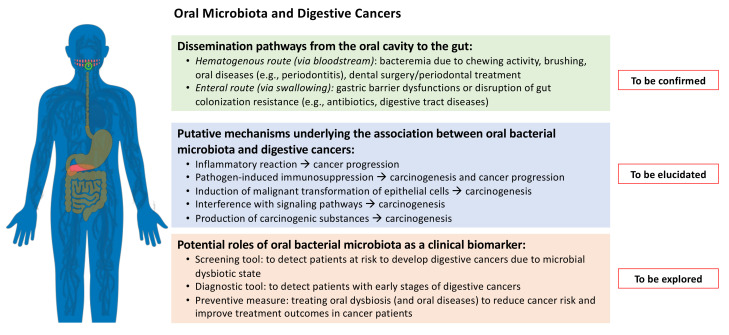
Schematic representation of the possible routes of oral bacteria transmigration from the oral cavity to the gut and the possible oral microbiota mechanisms in digestive cancers. The potential role of oral bacterial microbiota characterization for the screening and risk assessment of some types of digestive cancer is also described.

Finding the exact transmission pathways between the oral and the gut microbiota might provide evidence to support new methods for non-invasive cancer screening and could lead to control specific bacteria in their source, i.e., the oral cavity (Figure 2). Schmidt el al. [38] suggested an oral bacteria translocation in patients with CRC cancers. Flemer et al. [3] reported that oral swabs are associated with a sensitivity of 76% and a specificity of 90% in predicting colon adenomas; and a sensitivity of 98% and a specificity of 70% in predicting CRC. Sun et al. [50], found that salivary samples have a sensitivity of 97% and a specificity of 92% in predicting a GC, whereas Lu et al. [46] showed a sensitivity of 77% and a specificity of 78% for tongue coating to predict PC. Overall, the available studies suggest that a single oral bacterium has a limited ability to detect digestive cancer; conversely, multi-bacteria models have better performance and screening accuracy to differentiate patients with PC, EA, ESCC, GC, and CRC and to support the evaluation of the oral microbiota as a potential tool for prediction and prevention of digestive cancers [31]. Some evidence also supports a significant association between specific oral–gut bacteria and tumor stage, cancer-specific survival, and response to treatment [64,65,66,67,68], which, if confirmed and validated, would represent a novel and relevant strategy for reducing digestive cancer risk and improving cancer patients’ outcomes. Indeed, once the putative mechanisms of bacterial carcinogenesis can be elucidated and the role of oral microbiota precisely defined, its detection and characterization could be potentially used as a cancer biomarker and in the treatment of oral dysbiosis (e.g., periodontitis) as a preventive measure.

Due to their clinical relevance, these findings need to be validated in future studies with a large population of patients and reproducible protocols for oral microbiota sampling and analysis. The available literature is characterized by a high degree of heterogeneity in the methodology used. Moreover, the role of potential confounders, such as alcohol intake and smoking, which are known to drastically influence oral health and oral microbiota homeostasis [69,70], has been rarely considered. Alcohol abuse and smoking are also related to different cancers [70], but only a few of the studies selected in the present systematic review considered these factors in their analyses of the association between oral microbiota composition and digestive cancers [6,10,40,42,44,45,54,55,56]. Moreover, other confounders, such as oral hygiene habits [71], periodontal health status and severity of periodontitis, presence of other oral diseases, and dietary patterns [72], were disregarded. Tooth loss has been linked to an increased risk of GC development [73] and to an overall increased mortality rate [40]. Poor oral hygiene and tooth loss have also been linked to an increased risk of CRA occurrence [74,75]. Overall, these variables, which may influence oral dysbiosis and cancer development, need to be systematically considered in future research.

Another relevant issue to consider is the sampling method used in the different studies (e.g., saliva, tongue coating, oral rinse, subgingival plaque). The oral cavity consists of different sites (i.e., teeth, tongue, cheeks, hard and soft palates) that create specific niches for microbial colonization characterized by different oxygen levels, nutrient availability, and mechanical stress conditions. Consequently, distinct microbial communities can be found in the oral cavity according to the explored sites and the presence of diseases (e.g., periodontitis) [76]. The type of sample used could therefore affect the results while assessing the link between oral microbiota and cancer. Saliva represents the preferred sampling site to obtain oral microbiota DNA to process since it tends to reflect the microbiota from all oral sites and the associated disease [77]. Ryutaro et al. [78] showed no differences in microbiota diversity in samples of unstimulated saliva, stimulated saliva (through chewing a paraffin gum), and mouth rise water collected in specimen tubes. The authors concluded that mouth rise is a reliable sample for microbiota analysis and that it could be particularly useful in specific subsets of patients, e.g., in elderly patients or in patients with low salivary flow, often providing results comparable with pure saliva samples. Conversely, the study of Gomar-Vecher et al. [79] comparing unstimulated saliva collected on paper points and stimulated saliva collected after chewing paraffin gum showed significant differences in microbiota composition between the two sampling methods. Again, Ryutaro, J. et al. [78] showed a different microbiota composition in tongue coating samples collected by scraping the dorsum of the tongue with a specific specimen compared with saliva and mouth rise water samples. Xu, J. et al. [52] focused on the landmark flora of the four common tongue coatings in GC patients. The samples were collected from the middle section of tongue dorsum using a toothbrush and put into the test tube with saline. By describing the oral microbiota composition, the authors concluded that these data could be useful for future standardization of GC diagnosis based on tongue microbiota. Tongue coating is a common sampling method in Chinese medicine. However, its biological and molecular bases are not completely validated, and thus it is not a widespread sampling method in the Western world [80]. Han, S. et al. [39] showed a relationship between the risk of PC and the tongue coating microbiota collected by scraping the front and middle section of the tongue with a sterile spoon and put into a test tube with saline (repeated three times and centrifuged (2000 r/min) for 5 min). Finally, subgingival plaque can also be used. Theoretically, it reflects the local microbiome composition much more specifically than a salivary sample and should be preferred when simultaneously evaluating periodontal diseases. Nevertheless, this sample collection method is more invasive and requires qualified trained staff to harvest subgingival plaque [56,81]. Moreover, in the presence of periodontitis, great heterogeneity can be found in the microbiota composition of the subgingival plaque sampled in deep vs. shallow periodontal pockets or following periodontal treatments [82]. Kawasaki et al. [56] examined six common periodontal pathogens in the subgingival microbiota of patients with EC and compared them with the salivary microbiota using real-time PCR analysis. A stronger association was found for the microbiota sampled in subgingival plaque. Thus, methodological issues should be considered in the interpretation and generalization of the results [83].

The present systematic review has some limitations related to the type and quality of the pertinent literature considered. Overall, the body of evidence is of low-to-moderate quality, but the results support the need of further studies investigating the role of oral microbiota composition in digestive cancer patients. The qualitative synthesis of the literature highlights the need for standardization in study design and methods to obtain comparable results contributing to the emerging evidence and elucidating the current knowledge. Indeed, the literature is characterized by high heterogeneity related not only to methodological aspects (e.g., sample selection, microbiome preservation, collection methods, taxonomical assignments, DNA extraction methods, and statistical analyses) but also to population characteristics, lifestyle habits, and culture, which may influence oral microbiota composition. Indeed, geography, ethnicity, subsistence-specific variations in human microbiome composition [84], and the genetic risk of cancers [85] could influence the results. Moreover, while most of the studies included healthy controls from comparable populations of those of cancer patients, only two studies reported to have performed endoscopies in controls to rule out the presence of cancer or precancerous lesions. Globally, there is no evidence of external validity so far.

## 5. Conclusions

Despite the limited evidence available, the literature is consistent in suggesting a significant association between oral microbiota composition and digestive cancers. To date, it is not possible to identify specific bacteria species involved in digestive cancer development, but these findings support further research to characterize the oral microbiota of these patients. Indeed, future studies should analyze the possible microbiota dissemination patterns from the oral cavity and the rest of the digestive tract and the possible role of oral microbiota characterization as a screening tool for digestive cancers.

## Figures and Tables

**Table 1:** General characteristics of the selected studies investigating the association between oral microbiota and digestive cancers.

Reference	Country	Study Design	Study Sample Size (*n*)	Digestive Cancer Type	Oral Examination/Diagnosis
Farrell, J. et al. *Gut* 2012 [30]	USA	Matched case-control	103	PC	Not reported
Chen, X. et al. *PloS ONE* 2015 [53]	China	Case-control	235	ESCC	Number of teeth; Missing and filled teeth (MFT score), oral hygiene habits
Hu, J. et al. *Biomed*. *Res*. *Int*. 2015 [48]	China	Case-control	146	GC	No oral disease
May, X. et al. *J*. *Periodontol*. 2015 [35]	USA	Prospective cohort	1252	CRC	Periodontal examination, oral hygiene habits
Torres, P. et al. *Peer J*. 2015 [43]	USA	Case-control	108	PC	Not reported
Han, S. et al. *Int*. *J*. *Oncol*. 2016 [39]	China	Case-control	290	CRC and GC	Tongue examination; No oral disease
Kato, I. et al. *J*. *Epidemiol*. *Res*. 2016 [16]	USA	Population-based case-control	190	CRC	Not reported
Lu, H. et al. *Sci*. *Rep*. 2016 [57]	China	Matched case-control	60	LC	Full oral examination; No oral disease
Peters, B.A. et al. *Cancer Res*. 2017 [6]	USA	Case control	316	ESCC and EAC	Not reported
Olson, S. et al. *Cancer Causes Control*. 2017 [44]	USA	Case-control	137	PC, PDAC and IPMN	Number of missing teeth missing; periodontal diseases; number of dental visits in the past 10 years for checkup or cleaning; use of mouthwash at least once a week in the past 5 years
Fan, X. et al. *Gut* 2018 [45]	USA	Population-based nested matched case-control study	732	PC	Not reported
Flemer, B. et al. *Gut* 2018 [3]	Ireland	Case-control	234	CRC	Not reported
Russo, E. et al. *Front*. *Microbiol*. 2018 [36]	Italy	Case-control	20	CRC	Not reported
Sun, J. et al. *Oncol*. *Rep*. 2018 [50]	China	Case-control	50	GC	Not reported
Wu, J. et al. *J*. *Cancer* 2018 [51]	China	Case-control	137	GC	Not reported
Xu, J. et al. *Microb*. *Pathog*. 2019 [52]	China	Case-control	150	GC	Not reported
Lu, H. et al. *J*. *Oral Microbiol*. 2019 [46]	China	Case-control	55	PC	No oral disease
Schmidt, T. et al. *eLife* 2019 [38]	Europe	Case-control (Multicentric study)	520	CRC	Not reported
Yang, Y. et al. *Int*. *J*. *Cancer* 2019 [37]	USA	Nested matched case-control study	693	CRC	Oral health history
Wang, Q. et al. *Sci*. *Rep*. 2019 [54]	China	Case-control	41	ESCC	Oral health history
Guven, D.C. et al. *Biomark*. *Med*. 2019 [41]	Turkey	Case-control	148	CRC	Oral health history
Vogtmann, E. et al. *Cancer Med*. 2020 [42]	Iran	Case-control	558	PC	Not reported
Zhao, Q. et al. *Front*. *Cell*. *Infect*. *Microbiol*. 2020 [55]	China	Case-control	90	ESCC	Not reported
Zhang, S. et al. *Theranostics* 2020 [40]	China	Case-control	253	CRC	Not reported
Kawasaky, M. et al. *Cancer* 2020 [56]	Japan	Case-control	122	ESCC	Not reported
Li, D. et al. *Microb*. *Phatog*. 2020 [58]	China	Case-control	24	LC	Not reported
Wei, A.L. et al. *World J*. *Gastroenterol*. 2020 [47]	China	Case-control	114	PC	Not reported
Huang, K. et al. *Front*. *Cell*. *Infect*. *Microbiol*. 2021 [49]	China	Prospective cohort	293	GC	Not reported

Abbreviations: CRA stands for colorectal adenoma; CRC for colorectal cancer; EAC for esophageal adenocarcinoma; ESCC for esophageal squamous cell carcinoma; GC for gastric cancer; IPMN for intraductal papillary mucinous neoplasms; LC for liver cancer; PC for pancreatic cancer; PDAC for pancreatic ductal adenocarcinoma.

**Table 2:** Outcomes of the selected studies investigating the association between oral microbiota and digestive cancers.

Reference	Study Population Characteristics (*n*)	Sampling Method and Type(s) of Microbiological Analysis	Microbiota Associated with Cancer	Bacterial Quantification	Main Finding(s)
Farrell, J. et al. *Gut* 2012 [30]	*Discovery cohort*: - PC patients with resectable cancer (n = 10) - Matched healthy controls (n = 10) *Validation cohort:* - PC patients with resectable cancer (n = 28) - Matched healthy controls (n = 28) - Chronic pancreatitis patients (n = 27)	Saliva - Human Oral Microbe Identification Microarray - Real-time qPCR - 16S rRNA sequencing	- *Neisseria elongate* - *Streptococcus mitis*	Absolute amount	31 bacterial species/clusters were increased in the saliva of PC patients, and 25 bacterial species/clusters were decreased in comparison with healthy controls. Salivary microbiota may be a non-invasive biomarker.
Chen, X. et al. *PloS ONE* 2015 [53]	- ESCC patients (*n* = 87) - Dysplasia (*n* = 63) - Healthy controls (*n* = 85)	Saliva - 16S rRNA V3-V4 sequencing	- *Lautropia* - *Bulleidia* - *Catonella* - *Corynebacterium* - *Moryella* - *Peptococcus* - *Cardiobacterium*	Relative abundance	ESCC patients had a decreased microbial diversity compared with healthy controls and patients with dysplasia.
Hu, J. et al. *Biomed. Res. Int.* 2015 [48]	- GC patients with GC (*n* = 74) - Healthy controls (*n* = 72)	Tongue coating - 16S rRNA V3-V4 sequencing	- *Actinomyces* - *Streptococcus*	Relative abundance	Thick tongue coatings observed in GC patients presented lower microbial community diversity than thin tongue coatings of healthy controls.
May, X. et al. *J. Periodontol*. 2015 [35]	Buffalo Osteoporosis and Periodontal Disease Study cohort of postmenopausal females - CRC patients (*n* = 17) - Mean follow-up: 11.8 ± 3.8 y	Subgingival plaque - Indirect immunofluorescence	- *Fusobacterium nucleatum* - *Prevotella intermedia* - *Campylobacter rectus*(*borderline positive associations*)	Relative abundance	No associations were found between the presence of individual subgingival periodontal pathogens and the incident risk of cancer.
Torres, P. et al. *Peer J.* 2015 [43]	- PC patients (*n* = 8) - Patients with other pancreatic diseases or non-digestive disease/cancer (*n* = 78) - Healthy controls (*n* = 22)	Saliva - Real-time qPCR - 16S rRNA sequencing	- *Leptotrichia* - *Porphyromonas*	Relative abundance	Several bacterial genera differed in abundance between PC patients and controls. Bacteria abundance profiles in saliva may be useful biomarkers.
Han, S. et al. *Int. J. Oncol.* 2016 [39]	- CRC patients with CRC (*n* = 90) - GC patients with GC (*n* = 100) - Healthy controls (*n* = 100)	Tongue coating - 16S rRNA V3-V4 sequencing	- *Neisseria* - *Haemophilus* - *Fusobacterium* - *Porphyromonas* - *Prevotella*	Relative abundance	Tongue coating is thicker in cancer patients than in healthy controls. Six microorganisms at the species level were significantly different.
Kato, I. et al. *J. Epidemiol. Res.* 2016 [16]	- CRC patients (*n* = 68) - Healthy controls (*n* = 122)	Oral rinse - 16S rRNA V3-V4 sequencing	- *Lactobacillus* - *Rothia*	Relative abundance	No association between *Fusobacterium* abundance or presence and CRC.
Lu, H. et al. *Sci. Rep.* 2016 [57]	- LC patient with cirrhosis (*n* = 35) - Healthy controls (*n* = 25)	Tongue coating - 16S rRNA sequencing	- *Oribacterium* - *Fusobacterium*	Absolute amount	Significant microbial dysbiosis of tongue coats in LC patients.
Peters, B.A. et al. *Cancer Res.* 2017 [6]	- EAC patients (*n* = 81) - ESCC patients (*n* = 25) - Controls (*n* = 210)	Oral rinse - 16S rRNA sequencing	- *Tannerella forsythia* - *Streptococcus pneumoniae* - *Neisseria* - *Porphyromonas gingivalis*	Relative abundance	Differences in oral microbiota composition between cases and controls. Possible application for screening purpose.
Olson, S. et al. *Cancer Causes Control.* 2017 [44]	- PDAC patients (newly diagnosed) (*n* = 40) - IPMN patients (*n* = 39) - Healthy controls (*n* = 58)	Saliva - 16S rRNA sequencing	- *Firmicutes*	Relative abundance	PDAC cases did not differ in microbiota diversity from controls or IPMN patients.
Fan, X. et al. *Gut* 2018 [45]	From the CPS II and PLCO cohorts - PC patients (*n* = 361) - Controls (*n* = 371)	Oral rinse - 16S rRNA sequencing	- *Porphyromonas gingivalis* - *Aggregatibacter actinomycetemcomitans*	Relative abundance	Carriage of the periodontal pathogens and decreased relative abundance of *Fusobacteria* and its genus *Leptotrichia* are associated with subsequent risk of PC.
Flemer, B. et al. *Gut* 2018 [3]	- CRC patients (*n* = 99) - Patients with colorectal polyps (*n* = 32) - Controls (*n* = 103)	Oral swabs - 16S rRNA V3-V4 sequencing	- *Streptococcus* - *Prevotellas*	Absolute amount	A classification model of oral swab microbiota distinguishes individuals with CRC or polyps from controls.
Russo, E. et al. *Front. Microbiol.* 2018 [36]	- CRC patients (*n* = 10) - Healthy controls (*n* = 10)	Saliva - 16S rRNA V3-V4 sequencing - Real-time qPCR	- *Fusobacterium nucleatum* - *Bacteroides*	Relative abundance	Bacterial community composition differed significantly between CRC patients and healthy controls.
Sun, J. et al. *Oncol. Rep.* 2018 [50]	- GC patients (*n* = 37) - Healthy controls (*n* = 13)	Dental plaque and saliva - 16S rRNA V4 sequencing	- *Pseudomonadaceae* - *Dethiosulfovibrionaceae* - *Paraprevotellaceae* - *Veillonellaceae* - *Actinomycetaceae*	Absolute amount	There are differences in the biomass, species richness, and species diversity between GC patients and controls. A microbiome scoring system was designed to be a potential screening method for GC.
Wu, J. et al. *J. Cancer* 2018 [51]	- GC patients (newly diagnosed) (*n* = 57) - Healthy controls (*n* = 80)	Tongue coating - 16S rRNA V4 sequencing	- *Firmicutes* - *Bacteroidetes* - *Streptococcus,* - *Alloprevotella* - *Veillonella*	Relative abundance	Microbiome in tongue coating may have potential guiding value for early detection and prevention of GC.
Xu, J. et al. *Microb. Pathog.* 2019 [52]	- GC patients (*n* = 115) - Healthy controls (*n* = 35)	Tongue coating - 16S rRNA V4-V5 sequencing - 18S rRNA ITS1-2 region	- *Saccharibacteria* - *Prevotella* - *Acinetobacter*	Relative abundance	Richness and diversity of microbiome are not related to the variation of the four common types of tongue coating in GC patients.
Lu, H. et al. *J. Oral. Microbiol.* 2019 [46]	- PC patients (*n* = 30) - Healthy controls (*n* = 25)	Tongue coating - 16S rRNA V3-V4 sequencing	- *Haemophilus* - *Porphyromonas* - *Leptotrichia* - *Fusobacterium*	Absolute amount	PC patients are colonized by remarkably different tongue coating microbiota than healthy controls.
Schmidt, T. et al. *eLife* 2019 [38]	- CRC patients (*n* = 50) from the French colorectal cancer cohort - Healthy controls (*n* = 470)	Saliva - Metagenomic sequencing	- *Streptococcus Anginosus* - *Veilonella Atypica* - *Peptostreptococcus stomatis* - *Solobacterium Morrei*	Relative abundance	The oral cavity is an endogenous reservoir for gut microbial strains, with increased levels of transmission in CRC patients.
Yang, Y. et al. *Int. J. Cancer* 2019 [37]	- CRC cases (*n* = 231) - Controls (*n* = 462)	Oral rinse - 16S rRNA V4 sequencing	- *Treponema denticola* - *Prevotella intermedia* - *Actinobacteria* - *Bifidobacteriaceae* - *Prevotella denticola* - *Prevotella sp. oral taxon 300*	Relative abundance	Multiple oral bacterial taxa are associated with CRC risk.
Wang, Q. et al. *Sci. Rep.* 2019 [54]	- ESCC patients (*n* = 20) - Healthy controls (*n* = 21)	Saliva - 16S rDNA V3-V4	- *Actinomyces* - *Atopobium*	Relative abundance	Association between oral dysbiosis and risk of ESCC.
Guven, D.C. et al. *Biomark. Med.* 2019 [41]	- CRC patients (*n* = 71) - Controls (*n* = 77)	Saliva - Real-time PCR	- *Fusobacterium nucleatum* - *Streptococcus gallolyticus*	Absolute amount	Higher amounts of *Fusobacterium nucleatum* and *Streptococcus gallolyticus* in CRC patients.
Vogtmann, E. et al. *Cancer. Med.* 2020 [42]	- PC patients (*n* = 273) - Controls (*n* = 285)	Saliva - 16S rRNA V4 sequencing	- *Enterobacteriaceae* - *Lachnospiraceae* - *Bacteroidaceae* - *Staphylococcaceae*	Relative abundance	Increased levels of some oral bacteria and PC, with the overall microbial community different between PC patients and controls.
Zhao, Q. et al. *Front. Cell. Infect. Microbiol.*. 2020 [55]	- EC patients (*n* = 39) - Controls (*n* = 51)	Saliva - 16s rRNA V3-V4 sequencing	- *Firmicutes,* - *Negativicutes* - *Selenomonadales* - *Prevotellaceae* - *Prevotella* - *Veillonellaceae*	Relative abundance	Differences in oral microbiota composition between cases and controls.
Zhang, S. et al. *Theranostics* 2020 [40]	- CRC patients (*n* = 161) - CRA patients (*n* = 34) - Controls (*n* = 58)	Oral swabs - 16s rRNA V3-V4 sequencing	- *Fusobacterium* - *Treponema* - *Porphyromonas* - *Streptococcus* - *Faecalibacterium* - *Rothia*	Relative abundance.	Oral microbial composition and diversity were significantly different among the three groups, and the CRA group had the highest diversity.
Kawasaky, M. et al. *Cancer* 2020 [56]	- EC patients (*n* = 61) - Controls (*n* = 61)	Dental plaque and saliva - Real-time PCR	- *Bacteroides* - *Streptococcus* - *A. actinomycetemcomitans*	Relative abundance	Differences in oral microbiota composition between cases and controls. Stronger microbiota association with dental plaque sample. Possible application for screening purpose.
Li, D. et al. *Microb. Phatog.* 2020 [58]	- LC patients (*n* = 6) - Hepatitis B patients (*n* = 6) - Hepatitis B + Cirrhosis patients (*n* = 6) - Healthy controls (*n* = 6)	Saliva - 16S rRNA sequencing	- *Haemophilus,* - *Porphyromonas* - and *Filifactor*	Relative abundance	Difference in oral microbiota composition according to the different grade of disease.
Wei, A.L. et al. *World J. Gastroenterol.* 2020 [47]	- PC patients (*n* = 45) - Healthy controls (*n* = 69)	Saliva - 16S rRNA V3-V4 sequencing	- *Streptococcus* and *Leptotrichia*	Relative abundances	Differences in microbiota composition between cases and controls.
Huang, K. et al. *Front. Cell. Infect. Microbiol.* 2021 [49]	- GC patients (*n* = 99) - Patients with superficial gastritis (*n* = 101) - Patients with atrophic gastritis (*n* = 93)	Saliva - 16S rRNA V3-V4 sequencing	- *Streptococcus* - *Bifidobacterium*	Relative abundance	A distinct salivary microbiota was observed in patients with GC when comparing with SG and AG. Salivary microbiota could be used to predict GC as well as its non-malignant stages.

Abbreviations: CRA stands for colorectal adenoma; CRC for colorectal cancer; EAC for esophageal adenocarcinoma; ESCC for esophageal squamous cell carcinoma; GC for gastric cancer; IPMN for intraductal papillary mucinous neoplasms; LC for liver cancer; PC for pancreatic cancer; PDAC for pancreatic ductal adenocarcinoma.

**Table 3:** Quality assessment of the selected studies according to the star score of the Newcastle–Ottawa Scale, NOS, based on which * are assigned to three criteria, i.e., selection (with a maximum of 4 stars [****]), comparability (with a maximum of 2 stars [**]), and outcome (with a maximum of 3 stars [***]) for a maximum of 9 stars. Higher scores indicate lower risk of bias.

	Selection	Comparability	Outcome	Total Score
Farrell, J. et al. *Gut* 2012 [30]	***	*	**	6
Chen, X. et al. *PloS ONE* 2015 [53]	**	**	**	6
Hu, J. et al. *Biomed. Res. Int.* 2015 [48]	**	**	**	6
May, X. et al. *J. Periodontol.* 2015 [35]	**	**	***	7
Torres, P. et al. *Peer J.* 2015 [43]	****	*	**	7
Han, S. et al. *Int. J. Oncol.* 2016 [39]	****	*	**	7
Kato, I. et al. *J. Epidemiol. Res.* 2016 [16]	**	*	**	5
Lu, H. et al. *Sci. Rep.* 2016 [57]	**	**	**	6
Peters, B.A. et al. *Cancer Res.* 2017 [6]	**	**	**	6
Olson, S. et al. *Cancer Causes Control.* 2017 [44]	****	*	**	7
Fan, X. et al. *Gut* 2018 [45]	***	**	**	7
Flemer, B. et al. *Gut* 2018 [3]	**	*	***	6
Russo, E. et al. *Front. Microbiol.* 2018 [36]	**	*	***	6
Sun, J. et al. *Oncol. Rep.* 2018 [50]	****	*	**	7
Wu, J. et al. *J. Cancer* 2018 [51]	***	*	**	6
Xu, J. et al. *Microb. Pathog.* 2019 [52]	**	**	**	6
Lu, H. et al. *J. Oral Microbiol.* 2019 [46]	***	*	**	6
Schmidt, T. et al. *eLife* 2019 [38]	**	*	**	5
Yang, Y. et al. *Int. J. Cancer* 2019 [37]	**	**	**	6
Wang, Q. et al. *Sci. Rep.* 2019 [54]	**	*	**	5
Guven, D.C. et al. *Biomark. Med.* 2019 [41]	**	**	**	6
Vogtmann, E. et al. *Cancer Med.* 2020 [42]	**	**	**	6
Zhao, Q. et al. *Front. Cell. Infect. Microbiol.* 2020 [55]	**	**	**	6
Zhang, S. et al. *Theranostics* 2020 [40]	***	**	**	7
Kawasaky, M. et al. *Cancer* 2020 [56]	***	**	**	7
Li, D. et al. *Microb. Phatog.* 2020 [58]	**	**	**	6
Wei, A.L. et al. *World J. Gastroenterol.* 2020 [47]	**	*	**	5
Huang, K. et al. *Front. Cell. Infect. Microbiol.* 2021 [49]	**	**	**	6

## Data Availability

Data available upon request.

## Supplementary Materials

The following are available online at https://www.mdpi.com/article/10.3390/microorganisms9122585/s1, Table S1: PRISMA Checklist.
